# Detection of *APP* gene recombinant in human blood plasma

**DOI:** 10.1038/s41598-023-48993-7

**Published:** 2023-12-07

**Authors:** Shigeki Mitsunaga, Naoko Fujito, Hirofumi Nakaoka, Ryoko Imazeki, Eiichiro Nagata, Ituro Inoue

**Affiliations:** 1 Laboratory of Human Genetics, National Institute of Genetics, 1111 Yata, Mishima, Shizuoka 411-8540 Japan; 2 Department of Genetics, The Graduate University for Advanced Studies (SOKENDAI), Mishima, 411-8540 Japan; 3 Department of Cancer Genome Research, Sasaki Institute, Sasaki Foundation, Chiyoda-ku, Tokyo, 101-0062 Japan; 4 Department of Neurology, Tokai University School of Medicine, Isehara, Japan

**Keywords:** Molecular biology, Biomarkers, Diseases, Molecular medicine

## Abstract

The pathogenesis of Alzheimer’s disease (AD) is believed to involve the accumulation of amyloid-β in the brain, which is produced by the sequential cleavage of amyloid precursor protein (APP) by β-secretase and γ-secretase. Recently, analysis of genomic DNA and mRNA from postmortem brain neurons has revealed intra-exonic recombinants of *APP* (gencDNA), which have been implicated in the accumulation of amyloid-β. In this study, we computationally analyzed publicly available sequence data (SRA) using probe sequences we constructed to screen *APP* gencDNAs. *APP* gencDNAs were detected in SRAs constructed from both genomic DNA and RNA obtained from the postmortem brain and in the SRA constructed from plasma cell-free mRNA (cf-mRNA). The SRA constructed from plasma cf-mRNA showed a significant difference in the number of *APP* gencDNA reads between SAD and NCI: the *p*-value from the Mann–Whitney *U* test was 5.14 × 10^−6^. The transcripts were also found in circulating nucleic acids (CNA) from our plasma samples with NGS analysis. These data indicate that transcripts of *APP* gencDNA can be detected in blood plasma and suggest the possibility of using them as blood biomarkers for Alzheimer's disease.

## Introduction

Sporadic Alzheimer’s disease (SAD) is the leading cause of dementia. It is characterized by amyloid-β (Aβ) accumulation in plaques and subsequent or partially preceding accumulation of abnormally phosphorylated tau in neurofibrillary tangles^1,2^. Their accumulation causes neuronal damage, but the mechanism of pathogenesis is not fully understood. In addition, Aβ positron emission tomography (amyloid PET) has reported that Aβ accumulation begins as much as 20 years before the onset of dementia^3,4^. This long-term latent accumulation makes it difficult to diagnose dementia and mild cognitive impairment (MCI), a pre-dementia stage, in the early clinical phases.

Accumulation of Aβ is examined by amyloid PET, which is radioactive, expensive, and available at limited facilities. The main components of Aβ, Aβ42^5,6^, phosphorylated tau, and total tau^7,8^ have also been measured in cerebrospinal fluid (CSF) as biomarkers for SAD, but the CSF collection remains invasive. Therefore, research and development of blood biomarkers for early diagnosis and screening tests are also underway^9–13^. In addition to proteins/peptides measured in CSF, miRNAs^14,15^, long non-coding RNAs (lncRNAs)^16–18^, mRNA^19^, and circular RNA^20^ have also been studied. Attempts to use these various blood RNAs as biomarkers generally involve combining many RNA species. They are, therefore, somewhat complex.

Brain atrophy is observed with age but to a marked degree in SAD^21,22^. Atrophy of the amygdala, hippocampus, entorhinal cortex, and parahippocampal cortex is observed from early onset^23^. Based on differences in the degree of atrophy in SAD and no cognitive impairment (NCI), the prediction of progression from MCI to SAD using MRI imaging has also been studied^24–26^. The VSRAD, for example, analyzes regions of interest (ROI) in medial temporal structures such as the entorhinal cortex, hippocampus, and amygdala, where atrophy is more common in SAD patients.

On the other hand, genomic mosaicism due to somatic mutations exists in the brain and is observed in both SAD and NCI^27,28^. Somatic mutations include single nucleotide variants (SNVs)^29–32^, copy number variants (CNVs)^33,34^, aneuploidy^35^, and activation of retrotransposons^36,37^. These genomic mosaicisms have been observed in postmortem brains and have been difficult to use as biomarkers. Recently, genomic cDNAs of *APP* (*APP* gencDNA), one of the brain mosaicisms, and their transcripts were reported in the postmortem brain^38^, and another group has independently observed *APP* gencDNAs^31^.

*APP* gencDNA is formed by somatic recombination and integrated into the genomic DNA. It is characterized by the absence of introns and the presence of an intra-exonic junction. *APP* gencDNA is neuronal-specific, and the number of foci of *APP* gencDNA in DNA in situ hybridization (DISH) analysis is higher in SAD than in NCI. With age, an increase in DISH foci of *APP* gencDNA has also been reported using mouse models of SAD. In addition, the plasma nucleic acid levels have been reported to be increased in SAD compared to NCI^39^. Because brain atrophy is caused by necrosis/apoptosis^40,41^, these reports suggest that *APP* gencDNA and its transcripts are released into the plasma from injured neurons, as are other cfDNA and cfRNA. Since *APP* gencDNA is formed by recombination in somatic cells and not germline cells, its abundance is small, and it is considered that *APP* gencDNA in plasma is also very small. However, unlike proteins, nucleic acids are easily amplified, so it is possible that *APP* gencDNA could be detected even in plasma if it could be amplified. The concentration of high-mobility group box 1 (HMGB1) released from necrotic cells in CSF has also been higher in MCI than in SAD^40^. Furthermore, the release of *APP* gencDNA from injured neurons may reflect the state of injured neurons in the brain more than other blood biomarkers.

These considerations suggest that early diagnosis of SAD, even before the onset of dementia, may be possible if *APP* gencDNA in nucleic acids released into plasma in response to neuronal damage can be detected. Therefore, we first analyzed publicly available sequence data (sequence read archive, SRA) to confirm the presence of *APP* gencDNA and its transcripts. For this purpose, we constructed probe sequences assuming homologous recombination and performed a computational screening using the probe sequence. Specifically, after confirming that the probe sequences worked in the SRA associated with the publication in which *APP* gencDNA was reported, we analyzed published SRAs constructed from genomic DNA and mRNAs; both were obtained from postmortem brains and confirmed the existence of *APP* gencDNA in the brain. In addition, we also confirmed that transcripts from *APP* gencDNA were present in the published SRA constructed from cell-free mRNA (cf-mRNA) in plasma. We further performed NGS analysis to detect *APP* gencDNA and its transcripts using our plasma samples' circulating nucleic acids (CNA).

## Results

### Computational screening of SRA associated with the first report of *APP* gencDNA

**Figure 1:**
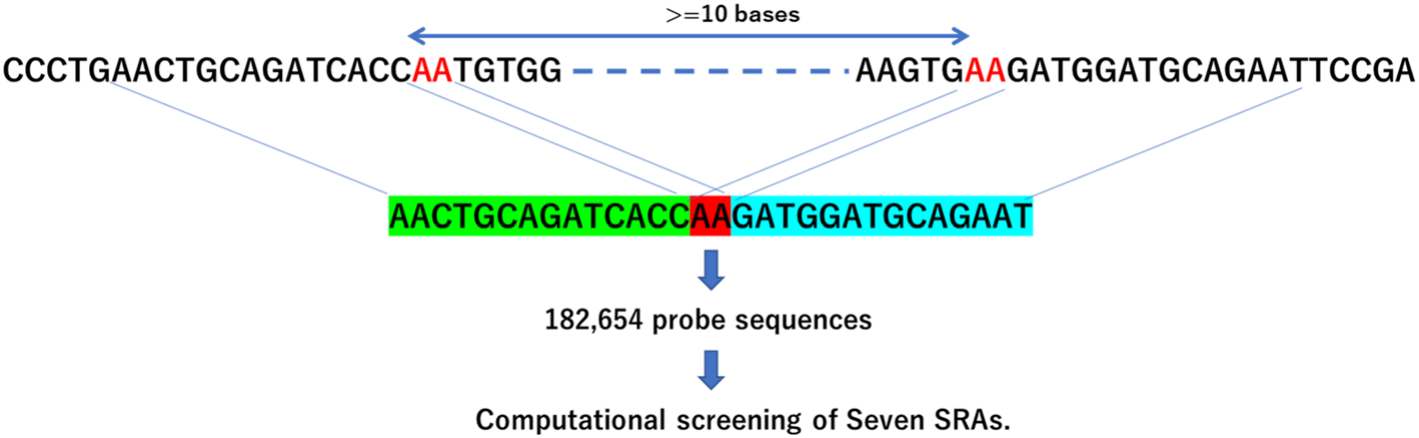
Construction of probe sequences for computational *APP* gencDNA screening. We searched for the same sequence of 2 bases at the 3′ end and 2 bases at the 5′ end. A probe sequence of 30 bases was created by combining 16 bases, including 2 homologous bases at the 3′ end and 16 bases including 2 homologous bases at the 5′ end, and removing one pair of 2 homologous bases. The probe sequence's construction region was the *APP* gene's coding region, and homologous regions at least 10 bases apart were selected. Probe sequences with identical sequences in the *APP* mRNA (APP transcript variant 1, Accession number NM_000484) and duplicates were removed, resulting in a final set of 182,654 probe sequences.

To confirm the presence of *APP* gencDNA and to get a complete picture of it, we designed probe sequences based on the *APP* mRNA sequence and screened publicly available sequence data (SRA) computationally. We created a total of 182,654 probe sequences assuming two-base homologous recombination (Fig. 1, Supplementary Table 1) (see “Construction of probe sequences and computational screening of SRAs” in “Materials and methods”). The constructed probe sequences were then used for computational screening of two runs, SRR7905478 and SRR7905479 (see “Analyzed SRAs in this study” in “Materials and methods”) of BioProject PRJNA493258, which were obtained by Pacbio-sequencing of the amplicon of the nested PCR of *APP* in postmortem human brain and associated with the first publication reporting the presence of *APP* gencDNA in postmortem human brain^38^.

The probe sequences observed in SRR7905478 and SRR7905479 were shown in Supplementary Table 2. Thirty-eight probe sequences were positive, and various intra-exonic junction sequences were detected. Three probe sequences, that is, three recombination sites, out of 38 probe sequences detected were common with the recombination sites reported in the first publication of *APP* gencDNA^38^. These results indicated that constructed probe sequences worked well for screening intra-exonic recombinant. The screening results are summarized in Table 1. The number of probe sequence positive reads for SRR7905479 in SAD cases was 190,934 out of 254,351 total reads; for SRR7905478 in NCI cases, it was 82,346 out of 360,290 total reads. Since these SRAs were constructed after nested PCR of *APP* amplifying between exons 1 and 18, general normalization using housekeeping genes was not possible, and the total read count normalized these read counts. The result was 0.751 for AD cases and 0.229 for NCI cases. These results indicate that *APP* intra-exonic recombination in SAD cases occurs more frequently than in NCI cases and are consistent with the previous reports^38^.

**Table 1:** Detection of APP gencDNA in the SRA of PRJNA493258.

BioProject	PRJNA493258	
Run	SRR7905479	SRR7905478
Tissue/sample	Neuron/genomic DNA	
Sequencing	Pacbio	
Enrichment	Nested PCR	
Disease	SAD	NCI
Number of cases*	5	5
Average total read/case	50,870	72,058
*APP* gencDNA**	38,187	16,469
Aβ-producible*	3334	53

*Mixture of 5 cases.

**Read count/case.

### Computational screening of SRAs constructed from genomic DNA and RNA of postmortem brains and plasma cf-mRNA

Having confirmed that the constructed probe sequences could detect *APP* gencDNAs, we next analyzed six SRAs constructed from genomic DNA or mRNA obtained from postmortem brains (Table 2). In SRAs constructed from genomic DNA using exon capture instead of nested PCR, *APP* gencDNA was hardly detected. However, *APP* gencDNA was indeed detected in SRAs constructed from mRNA. These results indicate that *APP* gencDNAs are certainly present and are transcribed in the brain, although their abundance is low.

**Table 2:** Detection of APP gencDNA in SRAs constructed from genomic DNA or mRNA of postmortem brains.

BioProject	PRJNA493258	PRJNA558504	PRJNA532465		PRJNA839035		PRJNA232669		PRJNA644383	
Run	SRR7905480	SRR9899152~4	SRR8898252~301							
Tissue/sample	Brain/genomic DNA				Brain/mRNA					
Sequencing	Illumina									
Enrichment	Capture-hybridization									
Disease	SAD	SAD	SAD	NCI	SAD	NCI	SAD	NCI	SAD	NCI
Number of cases	3	3	22	3	39	8	9	8	11	18
Average total reads/case	25,183,428	242,603,526	461,546,604	450,635,293	56,828,533	53,986,793	121,492,784	116,948,904	37,708,601	37,996,326
*APP* gencDNA*	2	10	0	0	2	2	11	12	6	4
Aβ-producible*	0	0	0	0	1	1	8	8	4	2

*Read count/case.

*APP* gencDNAs and their transcripts in the brain should be released extracellularly with apoptosis/necrosis, the cause of brain atrophy. And released those should emerge in peripheral blood. Therefore, we next analyzed SRA (PRJNA574438) constructed from cf-mRNA (CNA) in blood plasma (Table 3). Three hundred thirty-one probe sequences in total were detected in PRJNA574438 (Supplementary Table 3), two identical to those observed in SRR7905480 of BioProject PRJNA493258. Many probe sequence reads were detected in SRR7905478 and SRR7905479, constructed from nested PCR amplicons and associated with the publication in which APP gencDNA was first reported. Still, no positive probe sequences were common with them. In contrast, in the SRA constructed from postmortem brain mRNA, many positive probes were shared by SAD and NCI (Supplementary Table 3): the number of cases with positive probe sequences in PRJNA574438 was 125 of 127 for SAD and 96 of 115 for NCI.

**Table 3:** Detection of APP gencDNA in SRA constructed from cf-mRNA in blood plasma.

BioProject	PRJNA574438	
Run	SRR10192165~502	
Tissue/sample	plasma/cf-mRNA	
Sequencing	Illumina	
Enrichment	Capture-hybridization	
Disease	SAD	NCI
Number of cases	127	115
Average total read/case	22,777,062	17,823,100
*APP* gencDNA*	54	29
Aβ-producible*	52	29

*Read count/case.

### Comparison of the number of *APP* gencDNA reads in an SRA from plasma cf-mRNA

**Figure 2:**
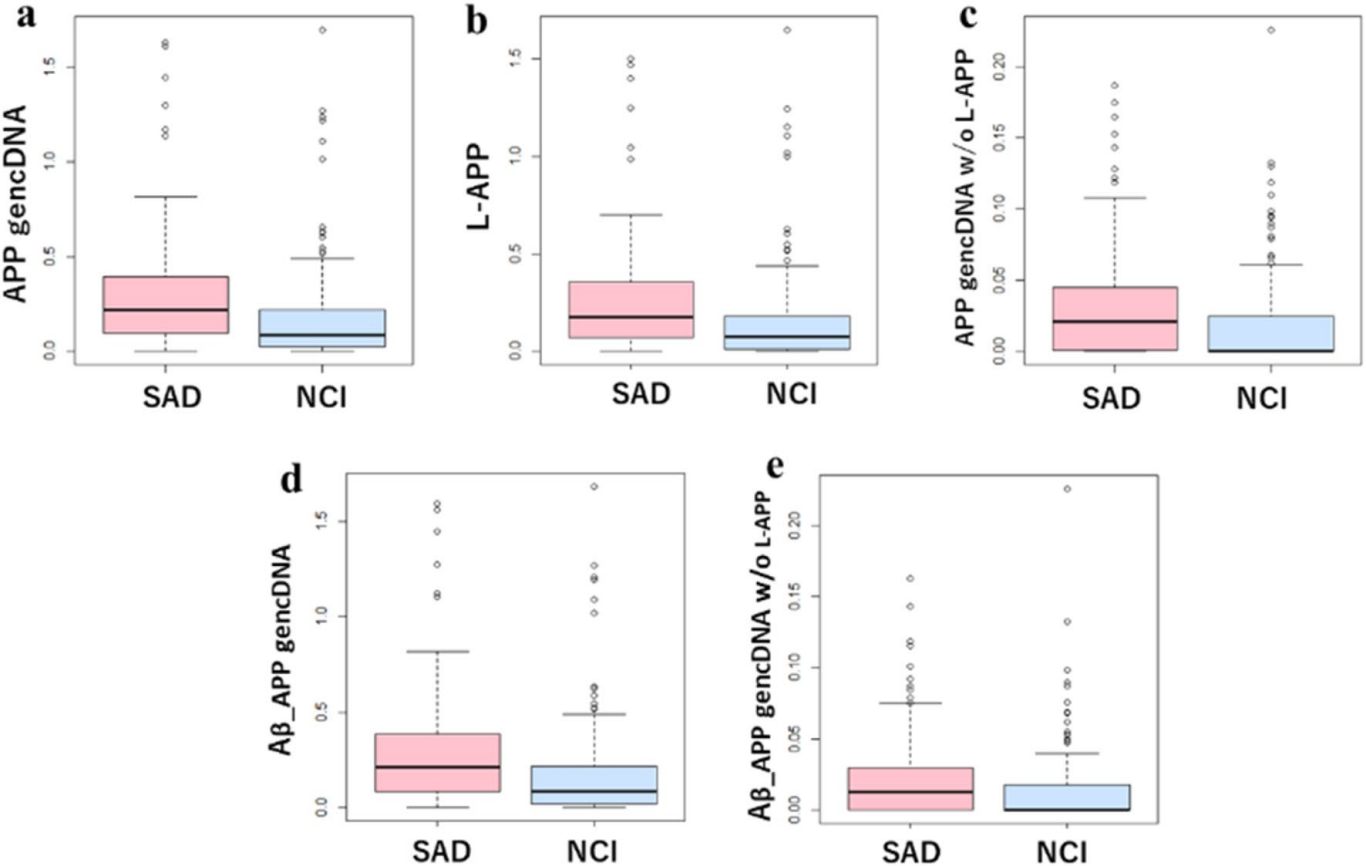
Comparison of APP gencDNA read counts in SAD and NCI. All read counts were normalized by GAPDH read counts. (**a**) Total read counts of APP gencDNA. (**b**) Read counts of L-APP. (**c**) Read counts of APP gencDNA minus L-APP. (**d**) Total read count of Aβ-producible APP gencDNA. (**e**) Total read count of Aβ-producible APP gencDNA minus L-APP. *p*-values by the Mann–Whitney *U* test are (**a**) 5.14 × 10^−6^, (**b**) 5.54 × 10^−6^, (**c**) 8.81 × 10^−5^, (**d**) 6.19 × 10^−6^, (**e**) 1.04 × 10^−3^.

When each read count of *APP* gencDNA read was normalized by dividing the read count of the housekeeping gene *GAPDH*, significant differences were observed between SAD and NCI: *p*-value by the Mann–Whitney *U* test was 5.14 × 10^−6^ (Fig. 2a). The distribution of read counts for the top ten probes in the positive cases was shown in Supplementary Fig. 1. For Aβ translation, frameshift did not occur in 207 of the 331 probe sequences (Supplementary Table 3). These reads were considered Aβ producible, but except for the probe sequence proseqff178928 positive, the number of other probe sequence positive reads was minimal. Table 4 shows the top 10 *APP* gencDNA-positive read counts, and Supplementary Table 3 shows all reads. Focusing on Aβ-producible reads, the average read count was 53/case for SAD and 33/case for NCI. On the other hand, no correlation between APP gencDNA normalized by GAPDH and MMSE score could be detected: for example, the *r*^2^ between L-APP and MMSE score was 0.0016. There was also no correlation between the number of Aβ-producing recombinants normalized by GAPDH and the MMSE score: *r*^2^ was 0.0056.

**Table 4:** Top 10 probe-sequence by read count in PRJNA574438.

Probe ID	Probe sequence	Junction	AB-producible	Number of positive case		Average read count per case	
			(no frameshift)	SAD	NCI	SAD	NCI
proseqff178928	AACACAGAAAACGAAGGTTCTGGGTTGACA	ex14:ex15	○	124	91	49.7	30.5
proseqff97091	CTGAGGTGGAAGAAGAAGAAGCCACAGAGA	ex6:ex6	○	21	11	0.4	0.2
proseqff93895	GAAGTAGCAGAGGAGGAAGAAGAAGAAGCC	ex6:ex6	○	17	6	0.2	0.1
proseqff90221	ATGCAGATGGGAGTGAAGAAGTGGCTGAGG	ex6:ex6	○	15	2	0.3	0.1
proseqff8330	CTCGGGCGCTGGAGGTCTACCCTGAACTGC	ex2:ex3	○	9	4	0.1	0.1
proseqff95093	GAGGAGGAAGAAGTGGCTGAGGAACCCTAC	ex6:ex6	○	11	5	0.1	0.1
proseqff184372	GAGCGCCACCTGTCCAGATGCAGAACTAGA	ex18:ex18		2	0	0.2	0.0
proseqff95821	GAAGAAGTGGCTGAGGAACCCTACGAAGAA	ex6:ex6	○	9	3	0.1	0.0
proseqff153526	AGCCAACGAGAGACAGACAGCACACCCTAA	ex11:ex12		1	0	0.2	0.0
proseqff102427	ATGGTGATGAGGTAGAGGAACCCTACGAAG	ex6:ex6	○	7	2	0.1	0.0

The most frequent probe sequence was proseqff178928, constructed as a recombinant at two bases homology region at the end of exon 14 and at the end of exon 15, which accounted for about 89% of the probe sequence positive reads. Its sequence was found to be identical to the junction sequence of exons 14 and 16 of the *APP* mRNA lacking exon 15, which is one of the *APP* isoforms and named L-APP mRNA^42^. L-APP mRNA is expressed in microglia and astrocytes^42^; it is not neuron-specific. Therefore, we conducted the Mann–Whitney *U* test by dividing probe sequence-positive *APP* gencDNA into proseqff178928-positive, L-APP, and the rest (Fig. 2b,c). Both groups showed significant differences between SAD and NCI: *p*-value 5.54 × 10^−6^ for L-APP and *p*-value for APP gencDNAs minus L-APP was 8.81 × 10^−5^. In addition, the Mann–Whitney *U* test, on the groups dividing according to their ability to produce amyloid-β, still showed significant differences between SAD and NCI (Fig. 2d,e): *p*-value 6.19 × 10^−6^ for APP gencDNAs including L-APP and *p*-value 1.04 × 10^−3^ for excluding L-APP.

*APP* mRNA with exon 8 spliced out is neuron-specific^43^. So, we compared the number of reads for the exon 7 and exon 9 junction sequences normalizing with *GAPDH* between SAD and NCI. The junction sequence of exons 7 and 9 is not included in the probe sequence we constructed because it does not contain a homologous region. The *p*-value was 2.73 × 10^−3^, which is significant but not a very small *p*-value.

### NGS analysis of circulating nucleic acids in blood plasma and comparison of other SRAs

To confirm the presence and detectability of *APP* gencDNA in plasma, we purified CNA from our plasma samples and performed Nanopore-sequencing using PCR products amplified with primer set in exon 1 and exon 18 of the *APP* gene (Supplementary Table 5). Although *APP* gencDNA could not be detected in some samples, a variety of *APP* gencDNA was detected in many samples (Supplementary Table 6). This analysis using our plasma samples also detected seven identical probes to those detected in SRAs constructed from plasma cf-mRNA (CNA); 37 probe sequences were shared between the SRA constructed from plasma cf-mRNA and the SRAs constructed from mRNA from postmortem brain (Supplementary Table 4). Many probe sequences were commonly found, suggesting that *APP* gencDNA formation may not have occurred randomly.

## Discussion

We could identify the reads with *APP* gencDNA sequences, that is, intra-exonic recombination sites, from several published sequence data (SRAs) using probe sequences constructed on the hypothesis of homologous recombination of two base duplications. Those are from the amplicon of a nested PCR of genomic DNA, enriched by capture hybridization of *APP* genomic DNA or mRNA from the postmortem brains. *APP* gencDNA sequences were detected in an SRA constructed from cf-mRNA in plasma, and we also detected them in circulating nucleic acid (CNA) in plasma by PCR amplification followed by Nanopore-sequencing using our plasma samples. Although the presence of *APP* gencDNA had been questioned^44^, these results suggest that *APP* gencDNA formed by intra-exonic recombination is undoubtedly present, its quantity is meager, detection requires a high degree of amplification, and transcripts of *APP* gencDNA are detectable in plasma. In addition, none of the APP intra-exonic recombinants detected in this study were identical to human sequences in all 30 base lengths, except for proseqff8330, in BLAST analysis. The proseqff8330 was identical to *APP* transcript variant 5, 6, cDNA FLJ50491 (AK294534), and APP639 (lacking exon 2, 7, and 8, expressed in fetal tissue and liver). These results indicate that the *APP* intra-exonic recombinants detected in this study are not derived from other genes.

A comparison of the number of reads of *APP* gencDNA transcripts containing L-APP in plasma normalized by *GAPDH* showed a significant difference between SAD and NCI. *APP* gencDNA transcripts in plasma are derived from damaged brain cells; since the accumulation of Aβ begins as early as 20 years before the onset of dementia and Aβ itself is cytotoxic, the release of *APP* gencDNA transcripts may start at a relatively early clinical stage, that is the pre-clinical stage, of Alzheimer's disease. The report that the extent of YAP-dependent necrosis is more pronounced during the MCI phase than after the onset of Alzheimer’s disease^40^ supports this hypothesis. In summary, *APP* gencDNA transcripts, including L-APP in plasma, may serve as blood biomarkers for Alzheimer's disease and detect the early clinical or pre-clinical stages of Alzheimer's disease.

The Mann–Whitney *U* test showed some outliers. Changes over time in biomarkers associated with the development of dementia are not linear changes but changes represented by a sigmoid curve. Neuronal injury, which may be related to the release of nucleic acids, is also characterized by a sigmoid curve^45^. That is the highest amount of released nucleic acids and YAP-dependent necrosis associated with the preclinical to early stages of Alzheimer’s disease but not with the dementia phase. Therefore, outliers in the Mann–Whitney *U* test are likely to be immediately after the onset of dementia in SAD and before the start of dementia in NCI.

Computational screening of *PSEN1* and molecular chaperones^46,47^ reported to be involved in the pathogenesis of SAD was performed using probe sequences constructed in the same manner as *APP*. Intra-exonic recombination was observed in the transcripts of several heat shock protein genes (Supplementary Table 7). Still, the number of read counts per case was lower than for *APP* transcripts (*APP* gencDNA), and no difference was observed between SAD and NCI: for example, *HSP90AA1* normalized with *GAPDH* sequence with a *p*-value of 0.639 by the Mann–Whitney *U* test. These results indicate that intra-exonic recombination is not *APP*-specific and consistent with another report^48^. Since reverse transcriptase activity is required for the formation of gencDNA^38^, intra-exonic recombinants are likely to be abundant in the brain, where activation of transposable elements occurs^49^. The low number of reads for gencDNA compared to the *APP* gencDNA also suggests that the contribution of PCR-mediated recombination^50^ to intra-exonic recombination of *APP* is minor.

In this study, proseqff178928, designed to detect sequences formed by homologous recombination between two bases at the 3′ end of exon 14 and two bases at the 3′ end of exon 15 of the APP gene, was the most frequently detected probe. And since the putative homologous recombination site detected by this probe is the same sequence as the junction site of exons 14 and 16 of L-APP, the *APP* isoform lacking exon 15, proseqff178928 could detect not only the *APP* gencDNA transcript but also L-APP. L-APP is expressed in astrocytes and microglia, which are involved in innate immunity in the brain, and these cells are also involved in removing Aβ plaques and aggregated tau proteins by activating and releasing inflammatory cytokines^51^. Since activated astrocytes and microglia cause programmed cell death (PCD) of them^51^, L-APP transcripts will be released from these dead cells. L-APP are expected to be more abundant than APP gencDNA because they are derived from normal genes, as opposed to transcripts derived from *APP* gene DNA, which are caused by somatic mutations. Therefore, the sequence that proseqff178928 was detecting was most likely derived primarily from L-APP, not from *APP* gencDNA. Astrocytes have been reported to upregulate glial fibrillary acidic protein (GFAP) when activated^52^. This GFAP is one of the blood biomarkers of Alzheimer's disease, and its blood levels are reduced by treatment with anti-Aβ antibodies^53^. Therefore, L-APP detected by proseqff178928 could be used as a blood biomarker for Alzheimer's disease and as a biomarker for therapeutic efficacy since it is derived from astrocytes as well as GFAP and is detected more frequently in SAD than in NCI.

## Materials and methods

### Analyzed SRAs in this study

Publicly available seven sequence-read archives (SRAs) were analyzed in this study: PRJNA493258^38^ (Tables 1 and 2), PRJNA558504^54^, PRJNA532465^31^, PRJNA839035^55^, PRJNA232669^56^, PRJNA644383^57^ (Table 2), and PRJNA574438^19^ (Table 3). SRR7905478 and SRR7905479 from PRJNA493258 were constructed by PacBio sequencing of the amplicon obtained from the nested PCR of *APP* using five normal human brains (SRR7905478) and five SAD patient brains (SRR7905479). SRR7905480 of PRJNA493258, PRJNA558504, and PRJNA532465 were constructed by exon capture hybridization and Illumina sequencing of genomic DNA obtained from the brain. PRJNA839035, PRJNA232669, and PRJNA644383 are the SRA of RNA-seq of brain mRNA. PRJNA574438 was constructed from cell-free messenger RNA (cf-mRNA) in the blood plasma of 127 SAD patients and 116 age-matched controls. There were 95 duplicates in this SRA, and one disease state was not identified. In the case of duplicates, the average read count was used in the analysis. The sequence data for which the disease state was not identified were excluded from the data analysis.

### Construction of probe sequences and computational screening of SRAs

Probe sequences for screening of intra-exonic junction in *APP* cDNA were constructed based on the mRNA sequence (*APP* transcript variant 1, Accession number NM_000484) as follows: since homologous recombination occurs between regions of homology of two or more bases, 14 bases upstream of a two-base homologous region, a two-base homologous sequence, and 14 bases downstream of a homologous region were combined. This procedure resulted in a 30-base probe sequence (Fig. 1); only the coding sequence of *APP* was targeted, and the distance between homologous regions was at least ten bases to reduce interference from repeated sequences such as two-base and three-base sequences. Duplications were eliminated, and probe sequences with identical sequences in the *APP* mRNA (*APP* transcript variant 1, Accession number NM_000484) were deleted, resulting in a final set of 182,654 probe sequences (Supplementary Table 1). These probe sequences can detect intra-exonic junctions of not only 2-base homologous sequences but also longer than 2-base homologous sequences. Using this set of probe sequences, each fastq file was screened by the bbduk command (sourceforge.net/projects/bbmap/): cat flistL.txt | while read I # flist: list of fastq file; do echo $i >> wc_SRA1; /home/bbmap/bbduk.sh -da in = ${i} outm = clean.fq ref = reference.fa rename = t k = 30; # reference.fa: multi fasta file of probes; grep -o @SRR clean.fq | wc -w >> wc_SRA1; cat clean.fq >> wc_SRA1; done. The extracted fastq files with probe sequences were analyzed in EXCEL. R software and packages (“exactRankTests”, “tidyverse” and “beeswarm”) were used for statistical analysis and figure drawing. Reads in which two or more of the same probe sequence were detected were considered derived from PCR artifacts and excluded from the count. This was more common in SRR7905478 and SRR7905479, which were constructed from nested PCR amplicons.

### SAD cases

All studies were conducted in accordance with the Declaration of Helsinki and after approval of the institutional review committees of two participating institutions: Institutional Review Board for Clinical Research, Tokai University (approval number 22R-180), and Ethical Review Committee on Medical and Biological Research Involving Human Subjects, National Institute of Genetics (approval number R2-14). After diagnosed to be SAD and obtaining written informed consent at the Tokai University School of Medicine, plasma specimens from 11 SAD patients were collected (Supplementary Table 8). All plasma specimens were collected with the RNA Complete BCT (Streck), and separated plasma was stored at − 80 °C until use.

### Sequencing analysis of circulating nucleic acid in blood plasma

Circulating nucleic acids were extracted from 1 mL of blood plasma using QIAamp Circulating Nucleic Acid Kit (QIAGEN) and eluted in 19 μL. Double-stranded cDNA was synthesized using 16 μL of eluate and LunaScript RT SuperMix Kit (NEB) in a 20 μL total reaction mixture. After PCR amplification (Supplementary Table 5) using all the cDNA solutions and Q5Hot Start High-Fidelity 2× Master Mix, the sequencing library was prepared with Native barcoding following ONT instructions. Nanopore-sequencing was performed using 30 ng library solution in 12 μL obtained from 16 plasma samples. The *APP* gencDNA sequences were extracted using probe sequences described above.

### Supplementary Information

Supplementary Figure 1.

Supplementary Tables.

## Data Availability

The datasets generated and analysed during the current study are available with links to BioProject accession number PRJDB16023 in the DDBJ BioProject database.
