# Supplementary material for: Detection of APP gene recombinant in human blood plasma
Source: Sci Rep. 2023 Dec 7;13:21703. doi: 10.1038/s41598-023-48993-7 (PMC10709617; doi:10.1038/s41598-023-48993-7)
Supplement: Supplementary file 1 — Supplementary Figure 1. [file 41598_2023_48993_MOESM1_ESM.docx]

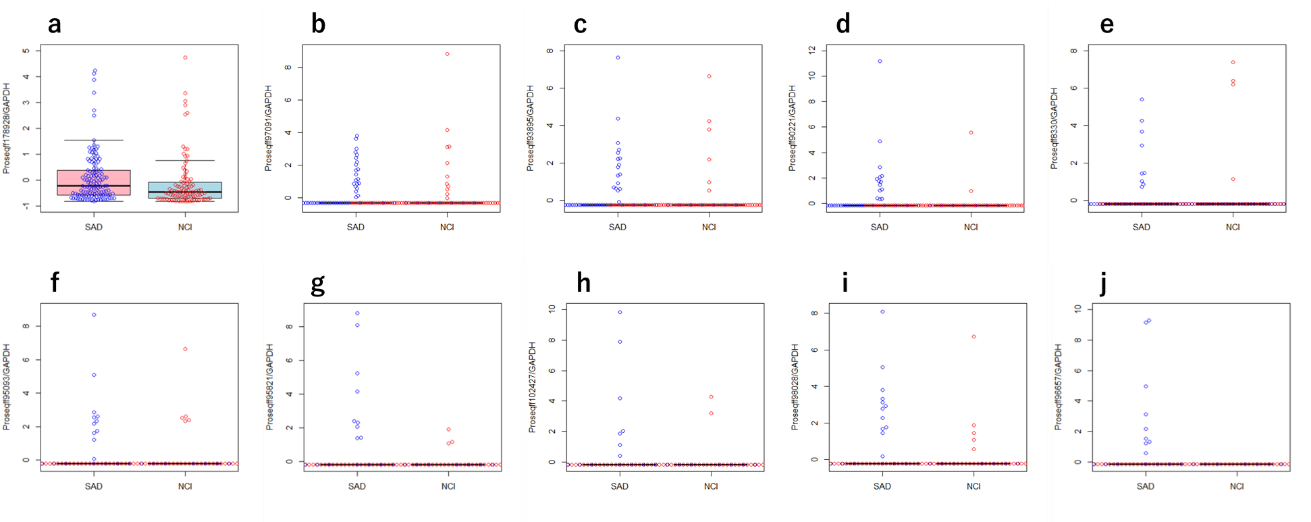


**Supplementary Figure 1. Read count distribution by the probe.** Read counts for the top ten probes in positive cases were plotted after dividing by GAPDH read count and Z-score normalization. a. proseqff178928, b. proseqff97091, c. Proseqff93895, d. Proseqff90221, e. Proseqff8330, f. Proseqff95093, g. Proseqff95821, h. Proseqff102427. i. Proseqff98028, j. Proseqff96657.
